# Sensor-based telerehabilitation system increases patient adherence after knee surgery

**DOI:** 10.1371/journal.pdig.0000175

**Published:** 2023-02-17

**Authors:** Jürgen Höher, Betty Lischke, Wolf Petersen, Natalie Mengis, Daniel Niederer, Thomas Stein, Thomas Stoffels, Robert Prill, Caroline Schmidt-Lucke

**Affiliations:** 1 Sportsclinic Cologne, Cologne, Germany; 2 Department for Orthopedics and Sports Traumatology, Merheim Hospital Cologne, University of Witten-Herdecke, Germany; 3 MEDIACC (Medico-academic Consultings), Berlin, Germany; 4 Martin Luther Hospital, Berlin, Germany; 5 Arcus Sports Clinic, Pforzheim, Germany; 6 Department of Sports Medicine and Exercise Physiology, Institute of Occupational, Social and Environmental Medicine, Goethe-University Frankfurt, Frankfurt am Main, Germany; 7 SPORTHOLOGICUM Frankfurt—Center for Sport and Joint Injuries, Frankfurt am Main, Germany; 8 OC Stadtmitte—Practice for Orthopedics & Surgery, Berlin, Germany; 9 Center of Orthopaedics and Traumatology, Brandenburg Medical School, University Hospital Brandenburg/Havel, Germany; The University of Sydney, AUSTRALIA

## Abstract

**Objectives:**

Implementing evidence-based recommendations with the option of patient-individualised and situation-specific adaptations in telerehabilitation may increase adherence with improved clinical outcome.

**Methods:**

As part of a registry-embedded hybrid design (part 1), digital medical device (DMD)-usage in a home-based setting was analysed in a multinational registry. The DMD combines an inertial motion-sensor system with instructions for exercises and functional tests on smartphones. A prospective, single-blinded, patient-controlled, multicentre intervention study (DRKS00023857) compared implementation capacity of the DMD to standard physiotherapy (part 2). Usage patterns by health care providers (HCP) were assessed (part 3).

**Results and conclusion:**

Registry raw data (10,311 measurements) were analysed from 604 DMD-users, demonstrating clinically expected rehabilitation progression post knee injuries. DMD-users performed tests for range-of-motion, coordination and strength/speed enabling insight to stage-specific rehabilitation (χ^2^ = 44.9, p<0.001). Intention-to-treat-analysis (part 2) revealed DMD-users to have significantly higher adherence to the rehabilitation intervention compared to the matched patient-control-group (86% [77–91] vs. 74% [68–82], p<0.05). DMD-users performed recommended exercises at home with higher intensity (p<0.05). HCP used DMD for clinical decision making. No adverse events related to the DMD were reported. Adherence to standard therapy recommendations can be increased using novel high quality DMD with high potential to improve clinical rehabilitation outcome, enabling evidence-based telerehabilitation.

## Author summary

Telerehabilitation refers to rehabilitation programmes delivered by electronic services such as medical apps. This technology can facilitate health care access and improve individual adherence to recommendations in a home-based setting, especially in the early phases of rehabilitation. We investigated key aspects of a sensor-guided medical app used for knee rehabilitation after surgery in three separate studies. We compared usage patterns, clinical effectiveness, and safety from 604 nationwide users and found that the sensor and medical app are able to measure correct values in the rehabilitation progress when used only by patients at home. Using the tests suggested by the app was safe. Patients with the app in the home environment were more likely to stick to recommendations of exercises given by doctors and physiotherapists and train harder compared to those only undertaking physiotherapy. Doctors and physiotherapist used sensor data and the medical app to decide what type of exercise a patient should perform. Our data show that telerehabilitation, and medical apps in particular, may become a game-changer for individualised and functional approaches to orthopaedic rehabilitation programmes.

## 1. Introduction

Telerehabilitation has a high potential to improve adherence and clinical outcomes in guideline-based rehabilitation programmes. Implementing evidence-based rehabilitation recommendations with individualised and situation-specific adaptations may increase adherence to health care providers’ recommendations at home with subsequent improved clinical outcomes [1]. In orthopaedics, telerehabilitation has so far not found entry to all suitable indication areas [1], despite first promising data on effectiveness and safety. The main expected benefits of telerehabilitation are saving resources, increasing scheduling flexibility and reproducibility of examinations, improving knowledge of the injury and rehabilitation, improving access to care, and increased engagement [2].

Further, telemedical applications may ensure correct performance of exercises. Validated sensors [3] that are linked to a telemedical application may provide a solution to reliably record and evaluate movements of the knee joint [4–6] to give immediate autofeedback. Digital medical devices (DMD, “medical apps”) need to be specifically designed to meet a special indications’ need [7] to ascertain continuous and high-quality rehabilitation e.g. after knee injury. Additional requirements are interoperability, robustness, consumer protection, usability, quality of medical content, patient safety, data safety and protection according to ISO 27001, as well as adherence to regulatory requirements.

In the initial phase of rehabilitation after acute knee joint injuries and/or after surgical interventions, active and passive knee joint mobility becomes relevant, while later functional stability, coordination, and strength become necessary before dynamic and speed exercises will be possible [8–10].

Adherence is the degree to which a person’s behaviour meets the agreed-upon recommendations of a health care provider [11]. There is a causal relation between adherence to guideline-compliant rehabilitation and clinical outcome [12–15]. Despite this knowledge, data of DMD-guided rehabilitation strategies to increase adherence are scarce.

We here present three different perspectives of the first DMD for knee rehabilitation. First, we sought to determine the usage patterns, measurement of clinical effectiveness and safety of the DMD in real-life from registry data. Next, we tested the prespecified hypothesis of its effect on clinical efficacy (adherence) and safety. Finally, we present the usage patterns and requirements of health care providers for a DMD as add-on to standard rehabilitation. All data stem from a registry-embedded hybrid design.

## 2. Methods

A registry-embedded hybrid design of DMD in rehabilitation in German-speaking countries in real life was performed. Approval was obtained by the local ethics committee prior to trial commencement (Ärztekammer Berlin, Eth-53/20). In the first part, a systematic data analysis of primary raw data of all users of a DMD-registry was conducted. This was followed by a prospective, single-blinded, patient-controlled, and formally multicentre study (part 2, “**OR**thelligent telematic rehabilitation **S**ystem **O**utco**ME**” study, ORSOME-study, DRKS00023857) on patients after surgical intervention for knee injuries. The prespecified primary endpoint of superiority of adherence to rehabilitation was quantified from a score deducted from a beforehand validated adherence questionnaire (ADREHA-score) and compared to a similar patient control group who received standard physiotherapy. In the third part, involved health care providers were requested to provide feedback on usage, adherence, and clinical outcome in patients using the telerehabilitation.

### 2.1. Orthelligent rehabilitation system

**Fig 1:**
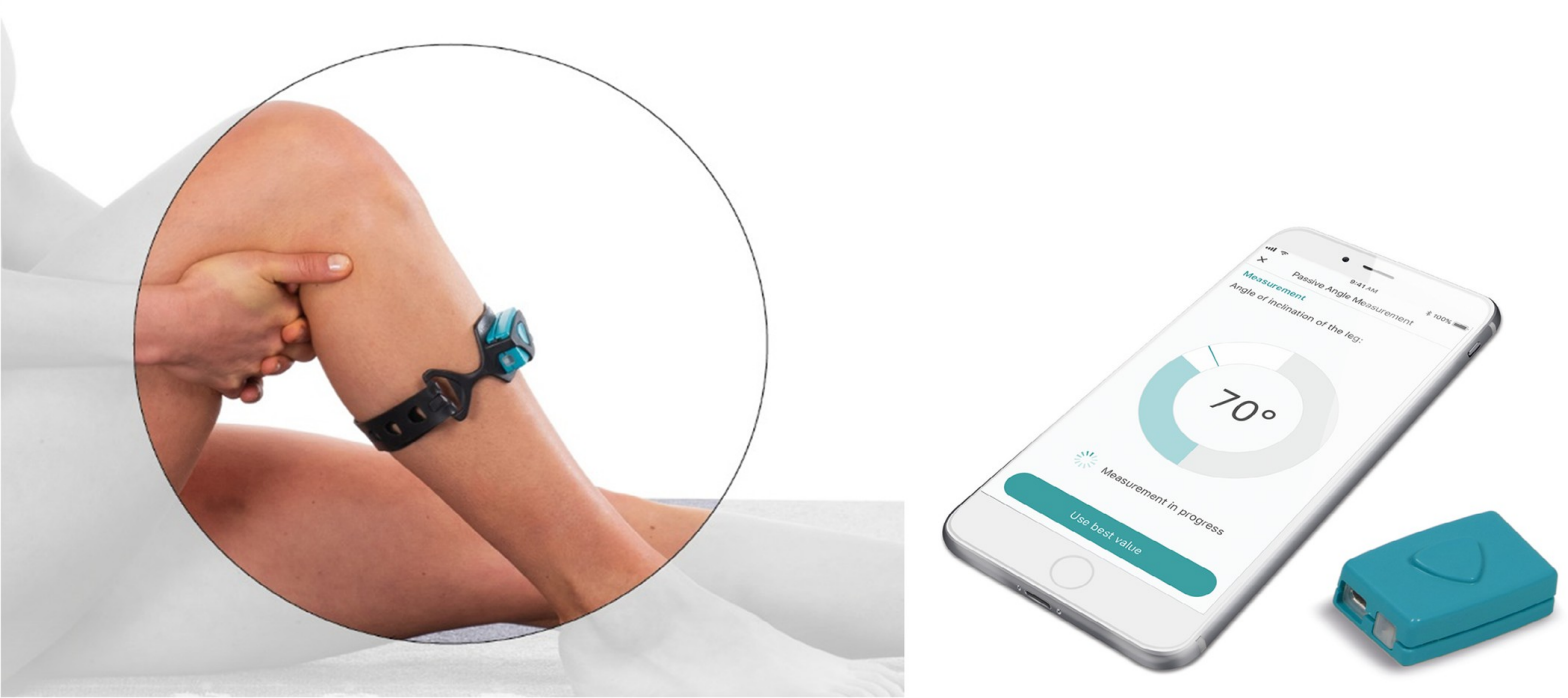
Demonstration of the digital medical device (DMD) and application.

The DMD (Orthelligent knee, OPED, Valley, Germany) is a validated Class I medical device [3]. It consists of instructions for exercises and functional tests (training schedule, paper, and online training videos for each exercise and parameters for training control for each exercise), an inertial motion sensor and a software to be downloaded on individual smartphones. Orthelligent provides stage-specific exercises in the course of rehabilitation for hip, knee and foot conditions (i.e., injuries or surgical interventions). The sensor of the DMD functions as an objective measurement instrument and is attached to the lower leg directly below the tibia head (Fig 1). The DMD is used in home-based settings as add-on to stage-specific standard physiotherapy after e.g. anterior cruciate ligament (ACL) reconstruction to perform specific tests in the categories range of motion (ROM, 3 tests), coordination (motor control, 2 tests), and dynamic tests (strength/speed, 2 tests) (for description of tests, see Table A in S1 File). All exercises and tests can be chosen individually by patients. Measured values of the affected leg are compared to the values of the contralateral unaffected leg and visualised as graphs demonstrating the relative values (symmetry or FIT-Index) and changes during the course of rehabilitation via the DMD-algorithm. The aim of the Orthelligent system is to motivate patients to perform the exercises defined by the health care provider sufficiently, frequently and qualitatively correctly, and to strengthen motivation through autofeedback in self-monitoring.

### 2.2. Registry data analysis (part 1)

From January 2018 to September 2020, 33,057 records of 604 patients who used a DMD-system for any indication were stored in a comprehensive multinational registry (Germany, Austria, Switzerland, DACH region). After plausibility-driven (range data control, testing frequency, outlier analyses) data cleanings, the remaining 10,311 values were further analysed. To exclude intra-week fluctuations and repetitively performed tests in order to record best individual measurements at acquisition dates, only the best value per week was considered for further analysis.

After performance of each test, demographic data, medical history, usage data, measurement results of each test performed, and information about sensation of pain on the injured leg were recorded. Pain was quantified by a visual analogue scale ranging from 0–10 (0 = no pain, 10 = worst pain imaginable) [16]. Information on demographic data, medical history, date of (planned) surgery, and all other disease-specific information was non-mandatory.

### 2.3. ORSOME study design (part 2 and 3)

Between December 2020 and February 2021, consecutive patients (n = 308) from the registry, who had received a knee orthosis, received a one-time invitation to participate in an online survey to give information on their individual 6 months rehabilitation after knee surgery. This procedure was in accordance with the corporate policy, EU-GPDR and individual consent to data usage and protection prior to using the DMD. Diagnosis data coded according to the International Classification of Disease (ICD) were available for all patients, and diseases were classified accordingly. 30 responded to the survey and gave their digital informed consent. The study was approved by the local ethics committee (Eth-53/20).

Inclusion criteria for analysis were: DMD-usage (≥ 1 test) or usage of knee orthosis treated within 6 months post-surgery, and informed consent. Exclusion criteria for analysis were: total and /or plausible answers <80%, or DMD-usage in the patient control group. The dataset retrieved from the prospective ORSOME-study contains all patients who made an assignment to one of the two treatment groups after surgical treatment of a knee joint injury or for knee joint damage or instability in an intention-to-treat-analysis (ITT).

### 2.4. Questionnaires

The following questionnaires were used:

- Medical history and preinjury level of sports activity: International Knee Documentation Committee (IKDC) forms (demographic, knee history, surgical documentation) [17]
- activity and return to activities of daily life: PAQ 50+ (subscales: housework, sport, occupation) [18]
- restrictions related to knee injury: ACL-RSI Knee Score (+ psychological component) [19] and subjective IKDC-form [20]
- QoL: KOOS QoL items [21]
- adherence: ADREHA (see S1 ADREHA Questionnaire)
- pain medication and adverse events: questionnaire.

All questionnaires were presented electronically with presentation of questions and layout identical to printed versions. Patients were requested to assess their conditions 6 months post-operatively, or if questionnaires were filled out prior to 6 months post-surgery (n = 4), give their actual condition and the time interval since intervention.

Health care providers (HCP) listed in the company’s data bank as prescribers or professional users of any of the company’s products received a one-time invitation to participate in an anonymised digital survey regarding the DMD. Fields of interest were: individual background, usage patterns, current and future indications, subjective assessment of the DMD, their judgement on area of application, perception of patients’ adherence and clinical outcomes and to document the benefits. All ratings by HCP were given on a scale from 0–10.

#### 2.4.1. Adherence questionnaire (ADREHA)

As there are no measurement instruments available for assessing adherence to rehabilitation for the target population of a cohort of patients undergoing rehabilitation after knee joint injuries or for chronic knee injuries in German-speaking countries and the corresponding cultural area, a standardised adherence score was derived from existing and validated components in German language. The questionnaire was tested and internally validated following the COSMIN guideline [22] according to a modified Delphi process [23] and used in electronic form. Elements of the ADREHA score were: (1) actual performance of home-based exercises, (2) type of recommendations, (3) actual performance of home-based exercises (frequency and duration), (4) intensity, convertibility, motivation and compatibility of exercises with daily life. For details of the process and calculation of the score see S1 ADREHA Questionnaire. To exclude whether socioeconomic status and initial intrinsic motivation might have distorted general adherence levels between the two groups, initial motivation and socioeconomic status taken from educational grade from sIKDC were compared.

### 2.5. Statistical methods

Data was collected and analysed in a GCP-compliant manner, in accordance with the ethical principles of the Declaration of Helsinki and in compliance with all EU regulations and German legislation.

Statistical analyses of the register data bank (part 1) were performed blinded from the database used for exploratory analysis. A range data plausibility check was performed for all independent and dependent outcomes; data was cleaned accordingly. Measurement results of the affected leg were analysed in the pre- and postoperative rehabilitation course for each test only for patients who provided their—planned or actual—date of intervention. Data of patients, who performed the corresponding test postoperatively with at least one repetition during the first 12 weeks, were evaluated for individual temporal courses. Missing or unclear values were not replaced. A minimum of 80% data completeness for key variables was prerequisite for subsequent analysis.

Data from the ORSOME study (part 2) were analysed in an ITT-analysis with the prespecified endpoint of increased adherence in the treatment group compared to the patient control group measured by the previously validated ADREHA-score. For details, please see S1 ADREHA Questionnaire.

For part 1–3, all continuous variables were examined for normal distribution using Q-Q plots. All metric data is non-normally distributed, presented as median with corresponding quartiles (median, [Q1-Q3]), and compared with Mann-Whitney-U tests. Bivariate non-parametric correlation (Spearman) was used to examine the internal validity of the individual questions used for the ADREHA-score in relation to each other and in relation to the ADREHA-score. Cumulative start to usage of the test categories during follow-up time was univariately evaluated by Kaplan-Meier analysis (log-rank test) and time to test for the earliest test within a category was chosen for analysis. Likelihood ratio chi-square (χ^2^) was used to estimate the relative chance of usage and 95% CIs are given. To analyse the frequency of use with respect to time to usage, “time to 50% of patients” (t50) and “time to 75% of patients” (t75) were analysed. Statistical significance is assumed if the null hypothesis can be rejected with a significance level of p ≤ 0.05. IBM SPSS Statistics (version 28.0.1, 2021) was used for analysis.

## 3. Results

The vast majority of the 604 patients from the register and the subgroup of patients in the ORSOME study arm had ACL reconstruction with or without concomitant knee injuries, as shown in Table 1.

**Table 1:** Demographic data of registry data and ORSOME-study.

	Registry data	ORSOME study		
		Total	DMD-users	Patient control group
**Number of patients**	604	27	17	10
**Gender m:f** Not specified	372:216 (63%:37%) 16 (0.03%)	12:15 (44%:56%) -	7:10 (41%:59%) -	5:5 (50%:50%) -
**Age** [years]	26-30 ^1^	30 [22-40]	31 [26-38]	26 [21-46]
**BMI** [kg/m^2^]	25 [22–27]	25 [22-28]	25 [21-28]	25 [24-27]
**Smoking status**	nd ^2^	2 (7%)	1 (6%)	1 (10%)
**Diagnosis [%]**				
ACL isolated	288 (48%)	10 (37%)	7 (41%)	3 (30%)
ACL with meniscus involvement	126 (21%)	9 (34%)	6 (35%)	3 (30%)
Combined ACL with other ligament involvement	22 (3%)	2 (7%)	2 (12%)	-
Isolated meniscus damage	25 (4%)	2 (7%)	2 (12%)	-
Lateral ligament damage	-	1 (4%)	-	1 (10%)
Other diagnoses ^3^	53 (9%)	2 (7%)	-	2 (20%)
Not specified	90 (15%)	1 (4%)	-	1 (10%)
**Treatment [%]**				
Surgical intervention	482 (80%)	27 (100%)	17 (100%)	10 (100%)
Not specified	122 (20%)	-	-	-
**Ethnicity [%]**	nd ^2^			
Caucasian		26 (96%)	16 (94%)	10 (100%)
Not specified		1 (4%)	1 (6%)	-
**Knee scores**				
sIKDC-Score		60.5 [52.8-74.5]	63.0 [53.3-73.0]	56.5 [49.0-82.5]
KOOS QoL		6.5 [4.0-10.3]	7.5 [4.0-10.0]	6.5 [2.8-11.8]
**Intensity of sports** **activity [%]**	nd ^2^			
Ambitious athlete		4 (15%)	3 (18%)	1 (10%)
Frequent sports activity		11 (41%)	6 (35%)	5 (50%)
Occasional sports activity		12 (44%)	8 (47%)	4 (40%)
No sports activity		-	-	-

^1^ median age group, age groups from < 16 years to > 51 years

^2^ nd = not determined

^3^ other diagnoses: registry data: Cartilage damage (n = 17), Posterior cruciate ligament (n = 15), Cartilage replacement procedure (n = 9), Knee prosthesis (n = 8), Patellar luxation (n = 3), Medial meniscus (n = 1), patient control group: Femoral paresis, Chronic knee joint damage (n = 1), Medial ligament rupture with bone tear (n = 1). Abbreviations: DMD, digital medical device; IKDC, International Knee Documentation Committee; KOOS, Knee Injury and Osteoarthritis Outcome Score

After plausibility-driven data cleanings in the registry, 91% of the values remained and after applying filters for multiple testing 10,311 records from all 604 patients remained for analysis, 69% of tests were recorded by repeated measurements on one day or within one week (for flowchart see Fig A in S1 File).

### General usage patterns of patients

DMD-tests were performed over individual periods of more than 16 weeks at home: 80% of the participants used the DMD before or after surgical intervention (15% started usage pre-operatively, n = 71, and 85% post-operatively, n = 411), whereas 20% (n = 122) of the DMD-users were treated only conservatively. Patients started using the DMD at three different time periods related to surgical interventions in home-based settings: (I) 1 year to 8 weeks pre-operatively, (II) peri-operatively (immediately pre- and up to 8 weeks post-surgery), and (III) >3 months post-operatively (see Fig B in S1 File). The majority (82%) of patients with preoperative start continued DMD-usage after their intervention.

Out of the 3 test categories of the DMD (range of motion, coordination and strength /speed), range of motion was used by nearly all patients (see Table 2).

**Table 2:** Proportion of patients who used Orthelligent preoperatively and postoperatively (n = 604).

	range of motion	coordination	strength /speed
Proportion of conservatively treated patients (n = 122)	91%	86%	68%
Proportion of patients performing tests pre-operatively (n = 71)	99%	77%	65%
Proportion of patients performing tests post-operatively (n = 469)	98%	73%	45%

### Time-dependent usage of test categories

**Fig 2:**
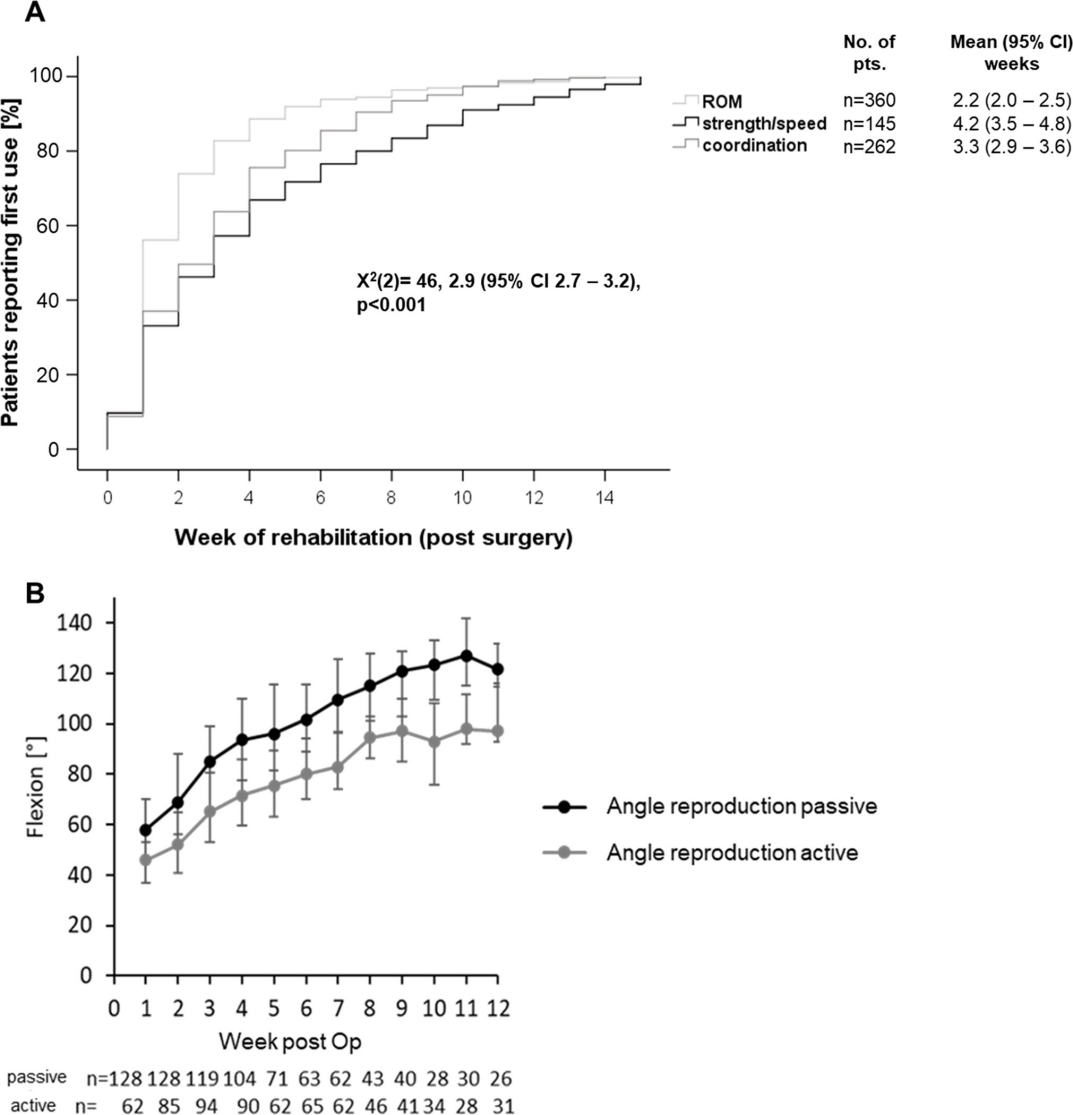
(A) Kaplan-Meier curve for begin of usage of the different test categories (ROM, coordination or strength/speed) for early rehabilitation (16 weeks) after knee surgery (n = 409, all patients using the digital medical device postoperatively). ROM, range-of motion; CI, confidence-interval; n, number of patients; (B) Postoperative course of passive and active knee joint flexion angles after surgical intervention measured with the sensor-based DMD (n = 128).

Patients started with tests for ROM in the earliest rehabilitation phase, followed by coordination after intervention, whereas strength / speed were started significantly later (χ^2^ 46 for group comparison, p<0.001, log-rank, Fig 2A). For detailed analysis of time-dependent usage of tests within the three test categories, please see Fig C in S1 File.

When regarding time intervals in which either 50% (t50) or 75% of patients (t75) had used single tests in the early rehabilitation phase (16 weeks), a much more differentiated pattern can be observed with regards to patient heterogeneity. For tests of ROM and coordination, t50 was 1 week and t75 was 2 weeks, similarly for coordination (t50: 2 weeks and t75: 4 weeks), indicating a rather homogenous rehabilitation progress for all patients for these tests. Interestingly, for the dynamic challenges (strength and speed) a different pattern (t50: 3 weeks and t75: 6 weeks) was observed, indicating that a small patient group had performed these tests safely at unexpectedly early timepoints. More than a quarter had not even performed dynamic tests up to 16 weeks.

### Quantification of joint mobility

Range of motion objectively quantified with the DMD increased during the course of postinterventional rehabilitation (6 weeks postoperatively active 80° [70°–94°] and passive knee flexion 102° [89°–116°], and 12 weeks postoperatively active 97° [93°–116°] and passive 122° [115°–132°], Fig 2B), as well as FIT-Index (LSI, Fig D in S1 File).

### Safety

Pain was reported in 12% of users at least to a moderate pain level (≥ 7 points on a 0–10 VAS), mainly for the dynamic tests (ROM 12%, coordination 13% and dynamic tests 27%) at the beginning and after 6 weeks (ROM 6%, coordination 7% and dynamic tests 18%). No adverse events were related to the DMD.

### Adherence to rehabilitation recommendations (Part 2)

Of 31 patients consented to participate in the survey regarding adherence to health care providers’ recommendations, 30 patients answered the questionnaires. After the exclusion of 3 patients (no surgical interventions), 27 data sets were available for analysis (for flowchart see Fig A in S1 File).

**Fig 3:**
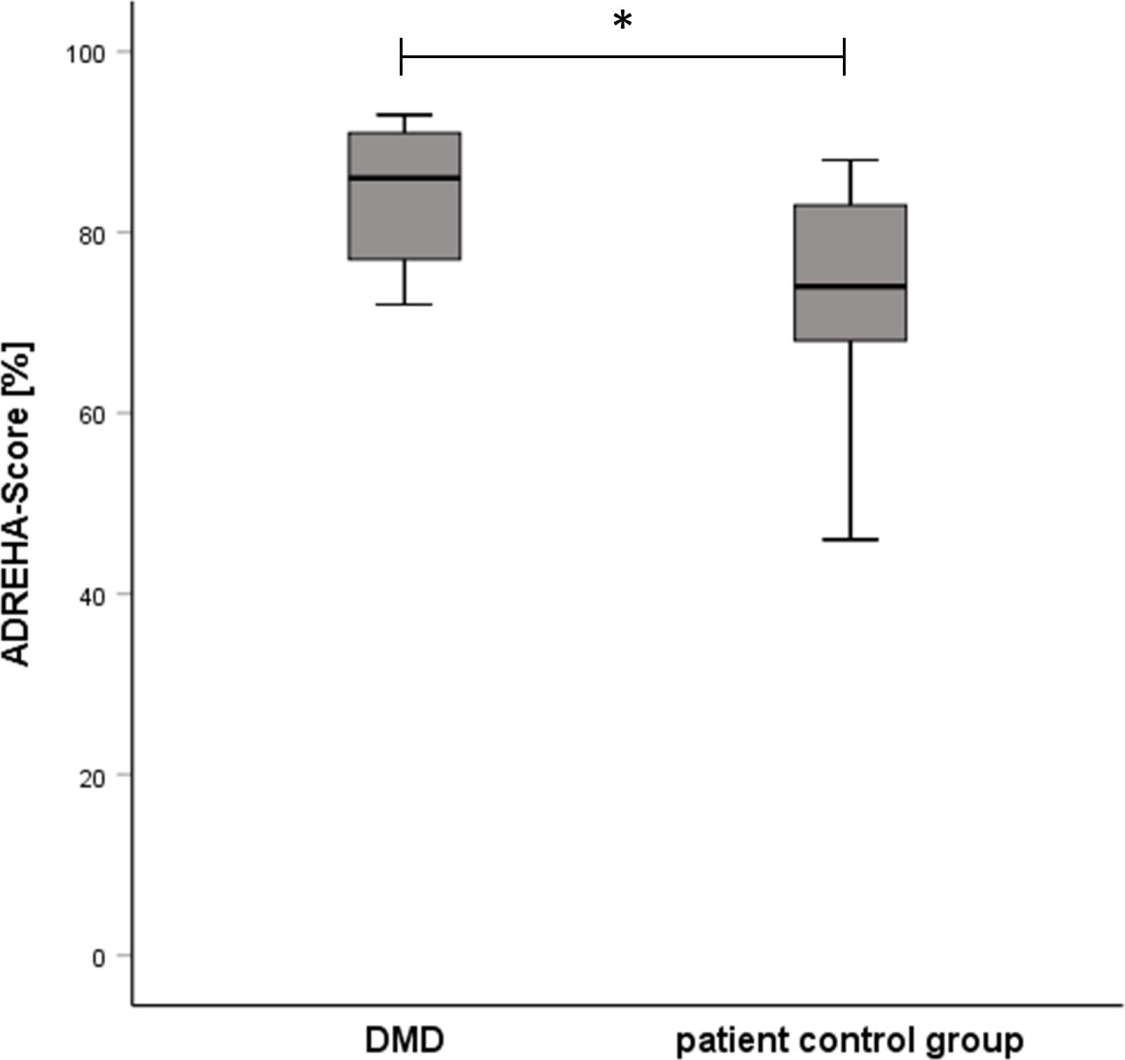
Adherence to health care providers recommendations regarding rehabilitation (ADREHA-Score in %) for DMD-users (n = 17, 86% [77–91]) and matched patient control group (n = 10, 74% [68–83]), * p<0.05.

Significantly higher adherence scores to the rehabilitation recommendations of health care providers in home-based DMD-users compared to the patient control group (ADREHA-Scores: 86% [77–91], 74% [68–83], p<0.05, and Fig 3) confirmed our prespecified primary endpoint. Socioeconomic status taken from educational grade from sIKDC and initial motivation was equal between DMD-users and patient control group (9.0 [6.0–10.0] vs. 8.0 [4.7–10.0]). Furthermore, DMD-users performed the self-exercises with higher self-reported intensities (8.0 [7.0–10.0], 5.5 [5.0–7.0], p<0.05) (see Table B in S1 File) and performed better in the other single components of the items contributing to the ADREHA-score, indicating that the combined adherence score was influenced by the total number of the selected items. For further details, please see S1 ADREHA Questionnaire.

The majority (13/17) of the DMD-users confirmed the benefit of monitoring their rehabilitation outcome as positive feedback to increase motivation. No adverse events related to DMD-usage in a cumulative 104 months DMD-usage period confirmed the safety of home-based use.

Making an appointment with their physical therapist was rated as rather hard (7 [5–10] on scale up to 10). Patients most frequently used their car (46%) or walked (35%) to the outpatients’ rehabilitation, requiring 5–15 minutes (65%), 16–30 minutes (31%), and up to 1 hour (4%).

### Health care providers’ usage patterns (Part 3)

The digital tool had been prescribed, recommended or used for rehabilitation by health care providers (HCP: physician consultants, n = 7, or physiotherapists, n = 15) for all joints of the lower extremity (knee: 95%, foot: 45%, hip: 18%) with appr. 20 (5–50) DMD-prescriptions / HCP during the last year. DMDs had been prescribed or recommended from adolescents (13–17 years) up to elderly (> 65 years) patients. The settings were postoperatively (48% of HCP), perioperatively (48% of HCP), and merely conservatively (4%) as home-based use, 43% had been for acute and 19% for chronic orthopaedic conditions, as well as for neurological (n = 3) or muscular disease (n = 6). 45% of HCP already used the ORS and 57% saw the potential for research to generate objective quantifiable data (physician consultants and physiotherapists alike) or as instrument to influence clinical decision making (90% of HCP, see also Fig E in S1 File).

### Health care providers’ independent accredited working mechanisms

The largest improvement of clinical outcome was seen through motivation 10.0 points [8.3–10.0], access to care 9.0 points [8.0–10.0], and athletic ambition 9.0 points [8.0–10.0] on a 10-point scale. Similarly, lack of patients’ motivation was confirmed as major risk factor by 86% of HCP for impaired clinical outcome. Greatest benefit of the DMD was seen—very similar to patients’ perception—in assessing patients’ rehabilitation status (100% of HCP), whether patients can return to work or sports (90% of HCP), optimising individual therapy schedules (81% of HCP), identifying further therapy needs (67% of HCP) and taking influence on patients’ rehabilitation (95% of HCP, see also Fig E in S1 File).

## 4. Discussion

This registry-embedded international hybrid study confirms the high potential of digital medical devices to increase the adherence to health care providers-prescribed rehabilitation after knee surgeries [24]. The medical device may even lead to better clinical outcomes than a non-instrumented standard rehabilitation (of the same therapy amounts). These findings are in line with a broad body of evidence of the social cognitive theory engaging patients to implement agreed-upon guidelines [25–33]. Treatment adherence was positively influenced by interventions to support self-directed physical rehabilitation of patients including elements to enhance self-efficacy and self-motivation, including direct autofeedback [34]. The sensor-based measurements are immediately visualised to the patient for guideline-recommended exercise categories to evaluate his/her training and the rehabilitation progress in a biofeedback manner.

The entire study population as well as the different subgroups were comparable regarding demographics, indication for rehabilitation and functional state, variables influencing adherence, representing the distribution of knee joint injuries in general and of the German population [35]. The sample is, thus, considered representative for the underlying population.

While the sole wearing of the local light weight sensor and the individual reading of the data corresponds to a computer technology ("wearables" or "wearable computers"), “Orthelligent” offers the additional possibility of intraindividual comparison (to the contralateral side, and over the course of time) and interindividual comparison to healthy individuals of an amateur or competitive level and to patients in different stages of rehabilitation.

Choice of different exercises and testing options of the DMD vary depending on the period before or after surgical intervention, or mere conservative treatment. The pattern presented here clearly shows that the individual tests of the Orthelligent are adequately used by patients at home, depending on their respective individual situation. Furthermore, data of this study support the relevance for an individualised and functional rather than time-dependent rehabilitation programme as currently implemented due to lack of large-scale real-world data. In conclusion, digital medical devices offer the opportunity to serve as a fact-based game-changer for optimal guidelines in rehabilitation programmes.

Digital medical device-usage was predominantly pain-free and safe in-home use. This is in line with similar results about the safety of other digitally supported rehabilitation programmes in orthopaedics [36,37]. One might speculate that the DMD had also been used as diagnostic tool in decision-making for the pre-operative rehabilitation guidance as shown by the frequent use over 12 to 3 months preoperatively and immediately before intervention in this cohort. Choice of exercises and tests post-reconstruction suggest that the DMD was used to monitor early and late rehabilitation phases and the possibility of return-to-sport relevant function (i.e.: are certain functional cut-offs fulfilled or not [24,38,39]). This form of utilisation is consistent with the function-based and graded rehabilitation in postoperative therapy after knee joint injury and surgery [8–10]. The exploratory analysis showed that the measurement of joint mobility corresponded to the values expected from clinical and scientific work with reliable and valid results and is thus suitable for objective quantification and measurement of functional status in clinical reality and for patients’ autofeedback [10,40]. Telerehabilitation should ideally facilitate access to the health care system when difficulties exist for reasons such as scheduling, staffing, distance or help needed from others. As seen in this study, DMD may overcome these hurdles.

Since the exercises guided by and the test values assessed by the DMD are not specific to rehabilitation after ACL reconstruction, it is not surprising to find health care providers applying the DMD for patients with acute and chronic conditions. The DMD was used predominantly for clinical indications for all joints of the lower extremity, for postoperative, conservative, or preoperative indications, and acute or chronic joint disease, as well as for neurologic or muscular diseases. Health care providers used the ORS in children, adolescents, adults, and also elderly patients (> 65 years). Furthermore, specialised service providers see the benefit of DMD and already use it for a larger group of indications, which goes far beyond the exemplary patient collective shown here [24].

The DMD with measurement functions used in this study offers the potential for a) objective quantitative monitoring of lower extremity function and rehabilitation progress, relevant for health care providers in clinic and research, b) decision making in adjusting rehabilitation measures or giving fact-based recommendations, or c) quality assurance of certain rehabilitation programmes. Planning and controlling the implementation of evidence-based rehabilitation steps are preconditions for individual function- and ability-based control of rehabilitation progress instead of rigid time-based rehabilitation protocols and would constitute a major step forward. This may lead to an adaptation of rehabilitation phases by supporting well-trained and proficient or hesitant and anxious patients. At the same time the risk of a too fast and therefore risky rehabilitation can be avoided, and the rehabilitation programme can be adapted to the individual needs / requirements.

We here present a new validated tool (ADREHA-score) for quantification of adherence to rehabilitation recommendations for the middle European culture in German, automatically calculating individuals’ adherence score. Further research is needed to test its validity for other indications and settings or its individual scoring in clinic or research.

### Strengths and limitations

The data set provided from the server of the DMD shares the strength and limitations of raw data sets primarily intended for non-scientific purposes. The data of more than 30.000 data sets from 604 patients used for originally intended use (post ACL-reconstruction rehabilitation in athletes) as well as other indications (neurological, chronic orthopaedic diseases, non-knee joints, pre-surgery or mere conservative treatment) and extended user circles (adolescents and the elderly) show the potential of DMD-usage as an add-on to standard physiotherapy in real life examined in a study population representative of the target population [24]. This finding was later confirmed in the survey amongst health care providers.

However, no assumptions can be made regarding individuals, who for any reason did not consider usage of this DMD suitable or helpful, or discontinued usage for any reason, be it discomfort or fast rehabilitation. Furthermore, response rate was relatively low in one study part. It is not clear from the evaluation whether and to what extent the patients may have already had pain prior to testing or, if applicable, whether testing was not performed in the case of pre-existing pain. Although designed as a prospective, blinded and controlled study with a prespecified primary endpoint and meticulous attention to confounding variables, an improved clinical outcome as a result to increased adherence can be deducted from clinical experience and is supported by the body of evidence. Still, the underlying causal relation has not been proven in this study and will need to be determined in a large-scale RCT considering sufficient numbers of different indications to confirm our first findings on increased adherence in a relatively small number of patients. Furthermore, prospective trials will be needed to clarify to which extent telerehabilitation might replace, essentially modify, or reduce the frequency of classic standard rehabilitation.

## Conclusion

Well-designed digital medical devices increase adherence to health care providers’ recommendations regarding rehabilitation programmes and are safe to use. Additionally, valid and reliable mobile measurement tools for quantification of complex functional movements offer substantial value for different health care provider groups. Implementing novel digital technologies may well be a key solution for functional rehabilitation protocols and programmes in the future.

## Supporting information

S1 File Table A: Description of execution of tests with the digital medical device. Fig A: Flowchart registry data analysis (part 1) and ORSOME study (part 2); n, number of patients; ITT, intent-to-treat. Fig B: Begin of use at three different time periods related to surgical interventions: (I) 1 year to 8 weeks pre-operatively, (II) peri-operatively (immediately pre- and up to 8 weeks post-surgery), and (III) >3 months post-operatively. Fig C: Kaplan-Meier curve for begin of usage of the DMD for rehabilitation within 16 weeks after knee surgery by test categories (A-C). CI, confidence-interval; n, number of patients. Fig D: Postoperative progression of FIT-Index after surgical intervention in 113 patients. Table B: ADREHA-Score, patient assessment of adherence score elements. Main components of ADREHA-score in arbitrary units (in %) on a scale of 0–10; median [IQR]. Fig E: Benefit of DMD for A) patients and B) health care providers from health care providers perception
(DOCX)

S1 ADREHA Questionnaire ADREHA Questionnaire. (DOCX)
