# Supplementary material for: Sensor-based telerehabilitation system increases patient adherence after knee surgery
Source: PLOS Digit Health. 2023 Feb 17;2(2):e0000175. doi: 10.1371/journal.pdig.0000175 (PMC9937459; doi:10.1371/journal.pdig.0000175)
Supplement: S1 File — Fig A: Flowchart registry data analysis (part 1) and ORSOME study (part 2); n, number of patients; ITT, intent-to-treat. Fig B: Begin of use at three different time periods related to surgical interventions: (I) 1 year to 8 weeks pre-operatively, (II) peri-operatively (immediately pre- and up to 8 weeks post-surgery), and (III) >3 months post-operatively. Fig C: Kaplan-Meier curve for begin of usage of the DMD for rehabilitation within 16 weeks after knee surgery by test categories (A-C). CI, confidence-interval; n, number of patients. Fig D: Postoperative progression of FIT-Index after surgical intervention in 113 patients. Table B: ADREHA-Score, patient assessment of adherence score elements. Main components of ADREHA-score in arbitrary units (in %) on a scale of 0–10; median [IQR]. Fig E: Benefit of DMD for A) patients and B) health care providers from health care providers perception (DOCX) [file pdig.0000175.s001.docx]

PDIG-D-22-00048

Sensor-based telerehabilitation system increases patient adherence after knee surgery

PLOS Digital Health

Supplemental Material

## Online Figures / Tables

Table A: Description of execution of tests with the digital medical device

|  | **Execution** | **Measurement parameters** |
| --- | --- | --- |
| 1. **Range of Motion (ROM)** | | |
| 1. **Angle measurement (passive)** | | |
| 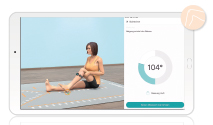 | Starting position in a seated position with legs extended and upper body upright. The measuring leg is pulled towards the body above the knee with the help of the arms. | Angle of inclination of the leg (°) |
| 1. **Stretch deficit** | | |
| 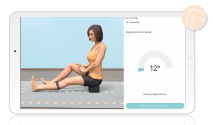 | Starting position in a seated position on a 10cm high foam plate with the upper body slightly bent and upright. The measuring leg is placed on a 15cm high foam roller. The foam roller is carefully pulled out. This is the starting position. The measuring leg is slowly lowered. | Angle of inclination of the leg (°) |
| 1. **Angle measurement (active)** | | |
| 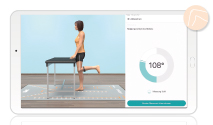 | Starting position in standing position, close to a table top, upper body is upright. Supporting the tabletop with the fingertips is permitted as an aid. The measuring leg is bent backwards as far as possible. | Angle of inclination of the leg (°) |
| 1. **Coordination** | | |
| 1. **Single leg stance** |  |  |
| 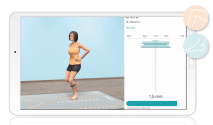 | Starting position in standing position, hands are supported on the hips, the upper body is upright. The non-measuring leg is bent backwards while the measuring leg is slowly bent. The position is held for 20s. | Lateral and medial movements (mm) |
| 1. **Angle reproduction** | | |
| 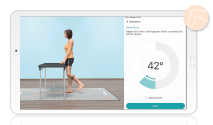 | Starting position in standing position, close to a table top, upper body is upright. Supporting the tabletop with the fingertips is permitted as an aid. A target angle is specified via the app, which is to be simulated with the measuring leg. There are three attempts to do this. The mean value of the three attempts is scored. | Difference to target angle (°) |
| **(C) Strength/Agility (Dynamic Testing)** | | |
| 1. **Vertical Jump** |  |  |
| 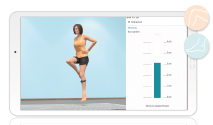 | Starting position in standing position, hands are supported on the hips, the upper body is upright. The non-measuring leg is angled upward (approx. 90°). With the measuring leg, a jump is performed from a standing position into the air. | Jumping height (cm) |
| 1. **Side Hop** |  |  |
| 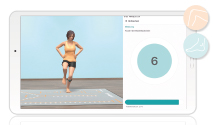 | Starting position in standing position, hands are supported on the hips, the upper body is upright. The non-measuring leg is slightly bent. With the measuring leg, so many jumps are performed from side to side within 30s. | No. of ground contacts |


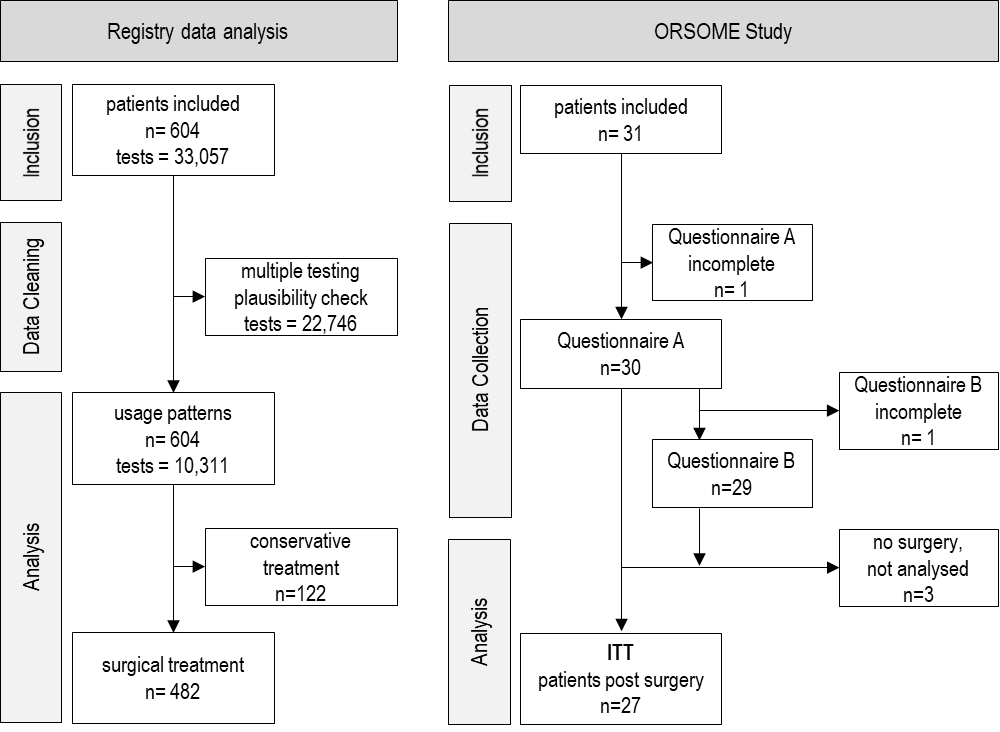


Fig A: Flowchart registry data analysis (part 1) and ORSOME study (part 2); n, number of patients; ITT, intent-to-treat.


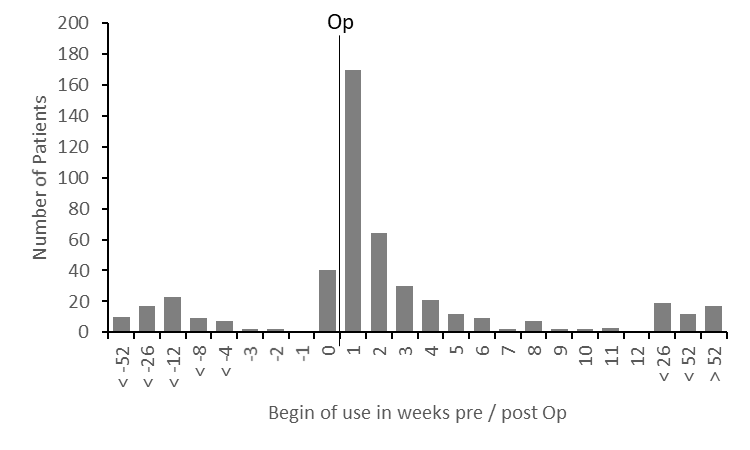


Fig B: Begin of use at three different time periods related to surgical interventions: (I) 1 year to 8 weeks pre-operatively, (II) peri-operatively (immediately pre- and up to 8 weeks post-surgery), and (III) >3 months post-operatively


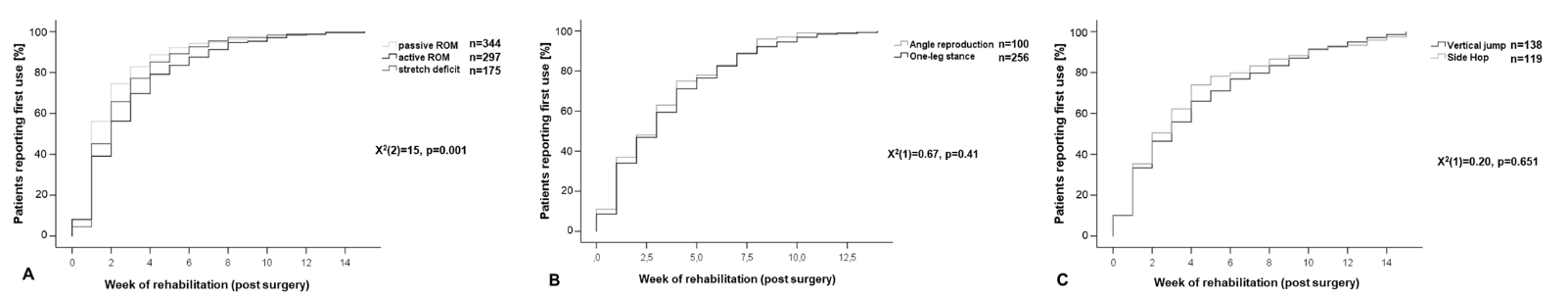


Fig C: Kaplan-Meier curve for begin of usage of the DMD for rehabilitation within 16 weeks after knee surgery by test categories (A-C). CI, confidence-interval; n, number of patients


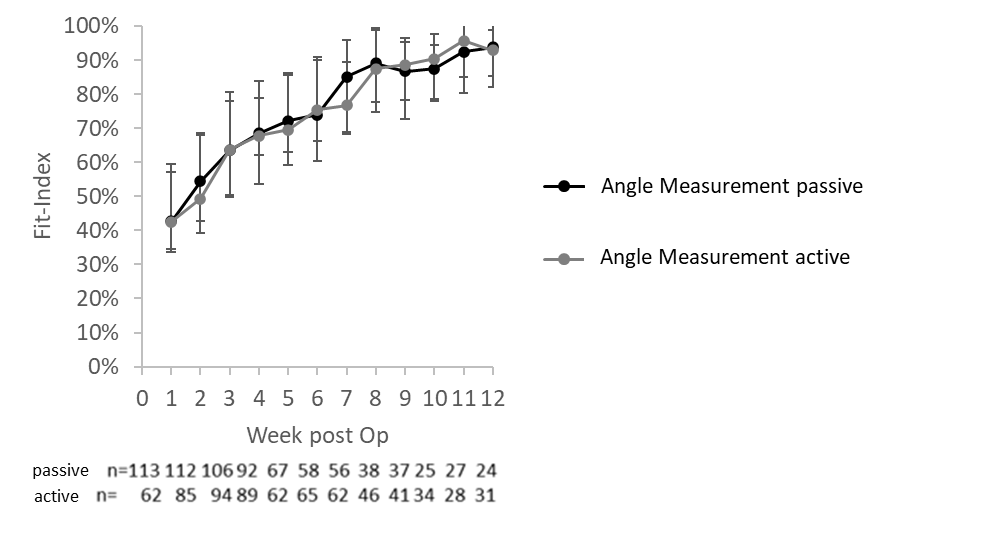


Fig D: Postoperative progression of FIT-Index after surgical intervention in 113 patients

Table B: ADREHA-Score, patient assessment of adherence score elements. Main components of ADREHA-score in arbitrary units (in %) on a scale of 0-10; median [IQR].

| **Variable** | **Total**  (N=27) | **DMD**  (N=17) | **Patient control group**  (N=10) | **p-value** |
| --- | --- | --- | --- | --- |
| ADREHA-Score | 80% [73 - 89] | 86% [77 - 91] | 74% [68 - 83] | 0.016 |
| **Main components of ADREHA-Score** |  |  |  |  |
| Intensity | 7.0 [5.0 - 9.0] | 8.0 [7.0 - 10.0] | 5.5 [5.0 - 7.0] | 0.011 |
| Convertibility of recommendations | 7.0 [5.0 - 9.0] | 8.0 [7.0 - 10.0] | 5.5 [4.0 - 8.0] | 0.075 |
| Motivation | 9.0 [5.0 - 10.0] | 9.0 [7.0 - 10.0] | 6.0 [4.0 - 9.0] | 0.092 |
| Compatibility of physiotherapy with daily life | 8.0 [4.0 - 10.0] | 8.0 [7.0 - 10.0] | 4.0 [2.0 - 9.0] | 0.022 |
| Compatibility of exercises at home with daily life | 8.0 [6.0 - 10.0] | 8.0 [8.0 - 10.0] | 6.0 [6.0 - 10.0] | 0.096 |

A B


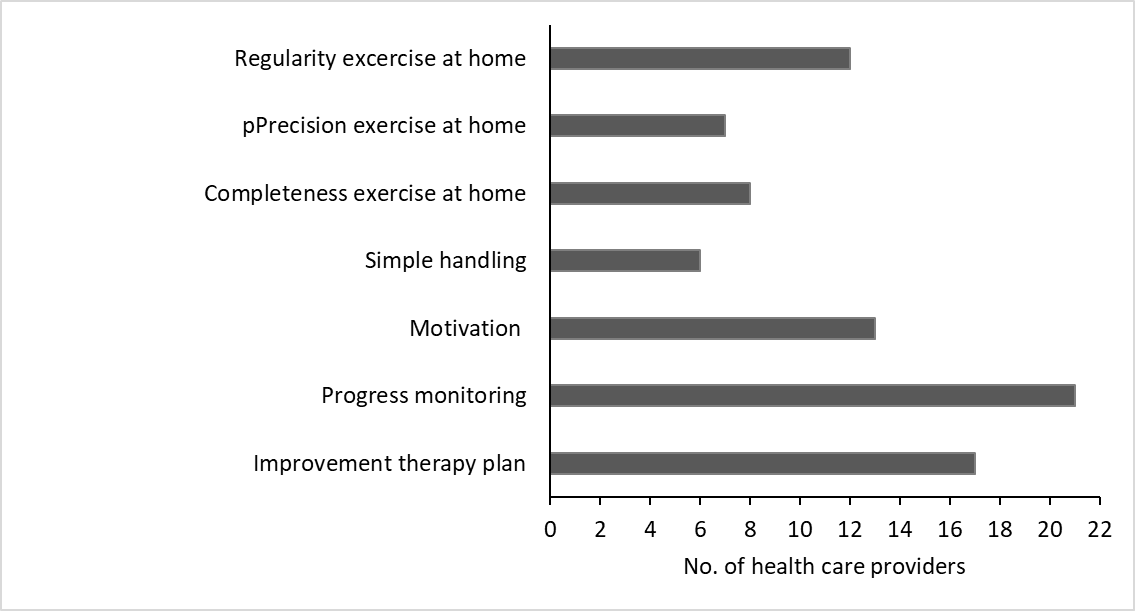

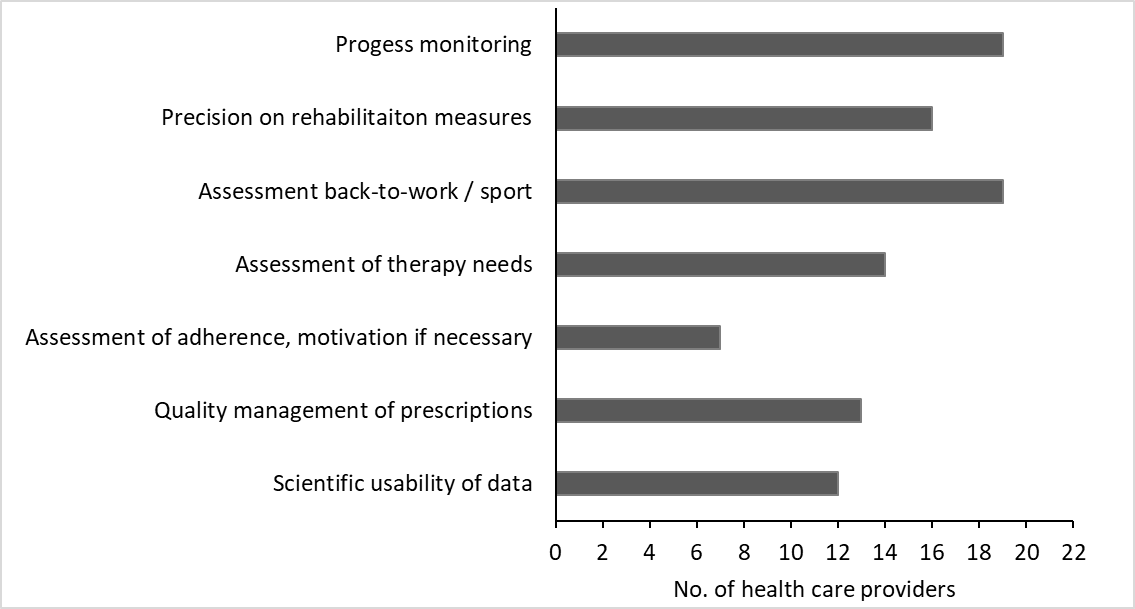


Fig E: Benefit of DMD for A) patients and B) health care providers from health care providers perception
