# Supplementary material for: Sensor-based telerehabilitation system increases patient adherence after knee surgery
Source: PLOS Digit Health. 2023 Feb 17;2(2):e0000175. doi: 10.1371/journal.pdig.0000175 (PMC9937459; doi:10.1371/journal.pdig.0000175)
Supplement: S1 ADREHA Questionnaire — (DOCX) [file pdig.0000175.s002.docx]

PDIG-D-22-00048

Sensor-based telerehabilitation system increases patient adherence after knee surgery

PLOS Digital Health

Supplemental Material

## ADREHA Questionnaire

**Generation of the adherence measurement instrument**

The questionnaires were developed according to the recommendation of an expert panel specialised in this field, consisting of physiotherapists (n=2) and orthopaedic specialists (n=2) and a statistician specialised in the development of questionnaires. For outpatient rehabilitation of adolescents and adults, the following parameters were considered relevant for the measurement of adherence to home exercises [25, 38] [39-42]: frequency, duration, intensity and type of exercise; on the patient's side their motivation, their goal, the general understanding of the execution of the exercises and the ease of implementation, integrability of exercises into everyday life, understanding of the necessity of the exercises and whether patients are able to perform the exercises in practice with sufficient frequency in addition to everyday life under supervision and with correction from physiotherapists. Therefore, the following elements were included in the calculation of the adherence score: Duration, frequency, intensity, motivation, integrability of physiotherapy in practice and integrability of self-exercises in everyday life and respective questions and statements formulated and respective scales assigned (see Online Table 3).

Subsequently, in a second round, the expert round was asked to suggest, delete, or add changes to the questions and statements. The changes were recorded and discussed in the panel together with a linguist and finalised by consensus. The questionnaire was then tested for comprehensibility and adapted on a heterogenous group of people with and without higher education qualification. In the then final version, these people were asked whether the questions and answer options were understandable, and to think aloud about their impressions about the questions and statements. The questionnaire was then checked for comprehensibility of instructions, programming and repeated at a time interval of 5 days to test for repeatability.

The questionnaires were then administered to a group of patients with knee joint injuries and patients with chronic knee injuries and indication for rehabilitation, who were not part of the confirmatory study population. They were previously asked whether they considered themselves to adhere to provider guidelines and asked to quantify this on a 5-point scale. This score was tested against that of the calculated adherence score (ADREHA-score). Finally, the ADREHA-questionnaire (see online suppl. Material 8.2) was sent to the patients of this confirmatory study as electronic version (ORSOME study). To optimise the quality of answers, in case answer options are not applicable or subjects don’t remember an item, this can be stated and the respective item will not be included in the calculation (see below). Overall, the relative frequency of missing data was 5.7% in this study with highest values for 7 to 12 (7%) and ≥ 12 months (8%).

Online Table 3: Components ADREHA Questionnaire

| **Components** | | **No of items** | **Question (English translation)** | **Response options** |
| --- | --- | --- | --- | --- |
| 1. | Performance self-exercises | 1 item | 1. Did you perform exercises independently at home after your surgery?   *Haben Sie nach Ihrer Operation Übungen selbständig zu Hause durchgeführt?* | yes / no |
| 2. | recommendations | 3 items | 1. Did you receive recommendations from your physician or physical therapist for own exercise at home?   *Haben Sie von Ihrem Arzt oder Physiotherapeuten Empfehlungen zur Eigenübung zu Hause erhalten?*   1. What were the recommendations? How long should each exercise session last?   *Wie sahen die Empfehlungen aus? Wie lange sollten die einzelnen Einheiten dauern?*   1. What were the recommendations? How often should you carry out an exercise session per week?   *Wie sahen die Empfehlungen aus? Wie häufig sollten Sie die einzelnen Einheiten pro Woche durchführen?* | yes / no 4 grades and 5 time intervals 4 grades and 5 time intervals |
| 3. | Conduct self-exercises | 2 items | 5. How long did each exercise session last that you carried out independently or with the help of the Orthelligent at home?  *Wie lange hat jede Übungseinheiten, die Sie eigenständig oder mit Hilfe des Orthelligent zu Hause durchgeführt haben, gedauert?*  6. How many exercise sessions did you carry out on your own independently or with the help of Orthelligent at home?  *Wie viele Übungseinheiten haben Sie eigenständig oder mit Hilfe des Orthelligent zu Hause durchgeführt?* | 4 grades and 5 time intervals 4 grades and 5 time intervals |
| 4. | Intensity | 1 item | 7. How intensively did you perform the exercises on your own?  *Wie intensiv haben Sie die Eigenübungen durchgeführt?* | 0-10 |
|  | Convertibility of recommendations | 1 item | 8. It was easy for me to carry out the exercises on my own according to the recommendations.  *Es fiel mir leicht die Eigenübungen entsprechend den Empfehlungen durchzuführen.* | 0-10 |
|  | Motivation | 1 item | 9. I was motivated to perform the exercises on my own.  *Ich war motiviert die Eigenübungen durchzuführen.* | 0-10 |
|  | Compatibility of exercises with daily life | 2 items | 10. The physiotherapy in the practice can be well integrated into my everyday life.  *Die Physiotherapie in der Praxis lässt sich gut in meinen Alltag integrieren.*  11. The exercises at home on my own can be well integrated into my everyday life.  *Die Eigenübungen zu Hause lassen sich gut in meinen Alltag integrieren.* | 0-10 |

**Calculation of the adherence score**

All answers are recorded electronically and the adherence score is automatically calculated. from a 11-item questionnaire. Elements are: 1. actual performance of home-based exercises (confirmation), 2. recommendations (confirmation, frequency and duration), 3. actual performance of home-based exercises (frequency and duration), 4. Intensity, convertibility, motivation and compatibility of exercises with daily life.

The score was calculated by comparing actual performance with recommendations (frequency and duration each to be included as one part of the total adherence score). By means of comparing recommendation and actual performance (elements 2 and 3), adherence was quantified derived from a matrix (see Online Table 4, Online Table 5) depending on level of deviation of actual performance from recommendation. Values were -2 to +2 points (5 increments) on a 29-point scale. For a 6-month rehabilitation period, 5 time-intervals were to be rated. For each time interval, adherence was scored divided by the number of time-intervals rated for inter-individual comparison in case not all 6 months were rated. Thus, adherence can be scored for different time intervals.

Certain definitions were prespecified: Patients who receive recommendations, but do not perform home-based exercises, are assigned the highest negative score (-2); patients who have not received recommendations or do not remember them, but perform self-exercises, the median recommendation of the group for the corresponding time interval is used as the basis for evaluation; patients who don’t remember to what extent they performed their own exercises, are not scored for that item; patients who don’t not performed any home-based exercises are assigned the lowest adherence level; since patients who by far exceeded their individual recommendations, are known to put themselves at risk for re-injury , received negative scores for that element, depending on the extend of over-exercising (see Online Table 4, Online Table 5).

To fill out the above-mention items, a certain amount of self-reflection is required. In order to get unreflected results from all individuals, we directly asked items of element 4 that relate to variables influencing adherence and are easy to answer. The extend, to which individuals rated their agreement to one of the 5 statements of element 4, was scored on a scale from 0 – 10. To compare these values to the above used scale (-2 to +2), the point values of the 5 statements were converted using a matrix (Online Table 6). For calculation of the adherence score, the implementation of frequency and duration from elements 1 to 3 are added to the 5 items of each of the 5 statements of element 4 to a cumulative value. The total cumulative value is then divided by the number of valid responses of the 2 parts (frequency and duration and the 5 statements) and given in percent to quantify the adherence level (ADREHA-score).

Online Table 4: Adherence questionnaire: Scoring of actual duration of rehabilitation

| Scoring duration [points] | | Recommendation | | | |
| --- | --- | --- | --- | --- | --- |
|  |  | < 5 min/ session | 5 - 20 min/ session | 20 - 40 min/ session | > 40 min/ session |
| Actual performance | < 5 min/session | 1 | 0 | -1 | -2 |
|  | 5 - 20 min/ session | 2 | 1 | 0 | -1 |
|  | 20 - 40 min/ session | -1 | 2 | 1 | 0 |
|  | > 40 min/ session | -2 | -1 | 2 | 1 |

Online Table 5: Adherence questionnaire: Scoring of actual frequency of rehabilitation

| Scoring frequency [points] | | Recommendation | | | |
| --- | --- | --- | --- | --- | --- |
|  |  | <=5 / week | 6-10 / week | 11-15 / week | > 15 week |
| Actual performance | <=5 / week | 1 | 0 | -1 | -2 |
|  | 6-10 / week | 2 | 1 | 0 | -1 |
|  | 11-15 / week | -1 | 2 | 1 | 0 |
|  | > 15 week | -2 | -1 | 2 | 1 |

Online Table 6: Adherence questionnaire: Scoring of intensity, convertibility, motivation and compatibility of exercises with daily life

| Point value | 0 | 1 | 2 | 3 | 4 | 5 | 6 | 7 | 8 | 9 | 10 |
| --- | --- | --- | --- | --- | --- | --- | --- | --- | --- | --- | --- |
| Score [points] | -2 | -1 | -1 | 0 | 0 | 1 | 1 | 1 | 2 | 2 | 2 |

**Internal validation of the ADREHA-score**

As shown in Online Table 7, there is a high intrinsic correlation of the individual variables with each other and with respect to the ADREHA-score. Not surprisingly, frequency and duration of exercise do not correlate with each other and the other variables. As a control, the correlation of the individual variables with the ADREHA-score was additionally calculated, in each case subtracting the corresponding variable (ADREHA-score minus independent variable). The significant correlations with the total ADREHA-score were thus be confirmed.

Since high intrinsic motivation is one of the strongest variables influencing adherence to rehabilitation [25] [26, 38-40, 43, 44] and also shows a high correlation with the ADREHA-score, we used this variable for further internal validation. Therefore, the entire group of patients - regardless of the use of the ORS - was dichotomised according to the median score for the question on motivation (median cut-off value: 90). The ADREHA-score was significantly higher in the group of self-esteemed more motivated patients than in the less motivated patients (88 [80 - 78] vs. 73 [68 - 91], p < 0.01). As a control, also for this calculation, the score of the question for motivation was subtracted from the ADREHA-score and this score was compared between the more and less motivated group, confirming the significant difference (p<0.01) in this modified adherence score between the two groups. These internal correlations and the described difference are a strong indicator of the validity of the ADREHA-score for measurement of adherence in rehabilitation.

Online Table 7: Internal correlation ADREHA-score

| **Adherence** | **Variable** | **Correlation coefficient (r)** | **Significance (p-value)** |
| --- | --- | --- | --- |
| ADREHA-score | intensity | 0.653 | ≤0.01 |
|  | convertibility | 0.064 | ≤0.01 |
|  | motivation | 0.725 | ≤0.01 |
|  | motivation and compatibility of physiotherapy with daily life | 0.506 | ≤0.01 |
|  | motivation and compatibility of self-exercises with daily life | 0.618 | ≤0.01 |
| ADREHA-score (-intensity) | intensity | 0.835 | ≤0.01 |
| ADREHA-score (-convertibility) | convertibility | 0.925 | ≤0.01 |
| ADREHA-score (-motivation) | motivation | 0.883 | ≤0.01 |
| ADREHA-score (-compatibility of physiotherapy with daily life) | motivation and compatibility of physiotherapy with daily life | 0.915 | ≤0.01 |
| ADREHA-score (-compatibility of self-exercises with daily life) | motivation and compatibility of self-exercises with daily life | 0.909 | ≤0.01 |
